# Bergamot Polyphenols Boost Therapeutic Effects of the Diet on Non-Alcoholic Steatohepatitis (NASH) Induced by “Junk Food”: Evidence for Anti-Inflammatory Activity

**DOI:** 10.3390/nu10111604

**Published:** 2018-11-01

**Authors:** Maddalena Parafati, Antonella Lascala, Daniele La Russa, Chiara Mignogna, Francesca Trimboli, Valeria Maria Morittu, Concetta Riillo, Rachele Macirella, Vincenzo Mollace, Elvira Brunelli, Elzbieta Janda

**Affiliations:** 1 Department of Health Sciences, Magna Graecia University, Campus Germaneto, 88100 Catanzaro, Italy; mparafati@unicz.it (M.P.); anto.lascala@gmail.com (A.L.); dlarussa@hotmail.it (D.L.R.); trimboli@unicz.it (F.T.); morittu@unicz.it (V.M.M.); criillo@unicz.it (C.R.); mollace@unicz.it (V.M.); 2 Interregional Research Center for Food Safety and Health, 88100 Catanzaro, Italy; 3 Department of Biology, Ecology and Earth Sciences, University of Calabria, 87036 Rende (CS), Italy; rachele.macirella@unical.it (R.M.); elvira.brunelli@unical.it (E.B.); 4 Department of Experimental and Clinical Medicine, Magna Graecia University, Campus Germaneto, 88100 Catanzaro, Italy; mignogna@unicz.it

**Keywords:** flavonoids, hepatic steatosis, cytokines, inflammation, nutraceutical treatment, food supplement

## Abstract

Wrong alimentary behaviors and so-called “junk food” are a driving force for the rising incidence of non-alcoholic fatty liver disease (NAFLD) among children and adults. The “junk food” toxicity can be studied in “cafeteria” (CAF) diet animal model. Young rats exposed to CAF diet become obese and rapidly develop NAFLD. We have previously showed that bergamot (*Citrus bergamia Risso et Poiteau*) flavonoids, in the form of bergamot polyphenol fraction (BPF), effectively prevent CAF diet-induced NAFLD in rats. Here, we addressed if BPF can accelerate therapeutic effects of weight loss induced by a normocaloric standard chow (SC) diet. 21 rats fed with CAF diet for 16 weeks to induce NAFLD with inflammatory features (NASH) were divided into three groups. Two groups were switched to SC diet supplemented or not with BPF (CAF/SC±BPF), while one group continued with CAF diet (CAF/CAF) for 10 weeks. BPF had no effect on SC diet-induced weight loss, but it accelerated hepatic lipid droplets clearance and reduced blood triglycerides. Accordingly, BPF improved insulin sensitivity, but had little effect on leptin levels. Interestingly, the inflammatory parameters were still elevated in CAF/SC livers compared to CAF/CAF group after 10 weeks of dietary intervention, despite over 90% hepatic fat reduction. In contrast, BPF supplementation decreased hepatic inflammation by reducing interleukin 6 (*Il6*) mRNA expression and increasing anti-inflammatory *Il10*, which correlated with fewer Kupffer cells and lower inflammatory foci score in CAF/SC+BPF livers compared to CAF/SC group. These data indicate that BPF mediates a specific anti-inflammatory activity in livers recovering from NASH, while it boosts lipid-lowering and anti-diabetic effects of the dietary intervention.

## 1. Introduction

Non-alcoholic fatty liver disease (NAFLD) is the most common liver disorder in Western countries, caused by fat and sugar-rich diet, sedentary life style and genetic predisposition [1,2,3]. The hallmark of NAFLD is excessive triglyceride (TGL) accumulation in the form of lipid droplets (LDs) in the cytoplasm of hepatocytes, which may be an isolated event (non-alcoholic fatty liver) or accompanied by evidence of inflammation and cell injury with or without fibrosis (non-alcoholic steatohepatitis, NASH). If untreated, NASH, the more aggressive form of NAFLD, may progress to cirrhosis and hepatocellular carcinoma [4].

NAFLD has become the leading cause of chronic liver disease among all age groups. Particularly alarming is the fast increase of adolescents and children diagnosed with NAFLD, reaching 10–20% in some studies [5,6,7]. Within the next 10 years, juvenile NAFLD is expected to become the most prevalent cause of liver pathology, liver failure and indication for liver transplantation in childhood and adolescence in the Western world [5,6,7].

The cause of rising incidence of NAFLD in children and adolescents is unclear, but it is believed that wrong alimentary behaviors, such as regular consumption of energy dense, highly refined and processed foods is a driving force for metabolic co-morbidities in young patients [8]. Such foods, rich in simple sugars, saturated and trans fats, and preservatives and poor in nutrients, prevalent in the Western diets, are commonly defined as junk food [8,9].

The effect of junk food can be modeled in experimental conditions by so-called cafeteria (CAF) diet, consisting of several favorite supermarket snacks of children such as sweet or briny cookies, milk chocolate, potato chips, processed meats, condensed milk, etc. [10,11]. Young laboratory animals exposed to CAF diet develop NAFLD as early as in two months which later degenerates into NASH [10,11,12]. This is associated with hyperglycemia and hypertriglyceridemia in rats.

Insulin-resistance (IR) is the central event in the pathogenesis of this disease, causing lipid overload in hepatocytes and lipid peroxidation. Since type 2 diabetes, obesity and dyslipidemia are the most prevalent associated co-morbidities, it has been proposed that NAFLD is the liver involvement of metabolic syndrome. The current FDA-approved medical recommendation for patients diagnosed with NAFLD is low-fat, fruit and vegetables-based diet and regular physical activity [4,13,14,15]. Indeed, dietary polyphenols, a large and heterogeneous group of phytochemicals in herbs and plant-based foods, have been associated with lower risk of NAFLD [16,17]. In particular, *Citrus* flavonoids have been shown to exert several positive health effects on glucose and lipid metabolism in experimental models [12,16,18,19,20,21,22] and humans [23,24,25]. 

In particular, bergamot is an endemic plant of Calabria region (Italy) belonging to the Rutaceae family [26]. Bergamot has attracted considerable attention due to its peculiar composition and high content of flavonoids, some of which are also found in other *Citrus* species [18,27]. BPF is a highly concentrated extract of glycosylated flavanones (naringin, neoeriocitrin and hesperidin) and flavones (diosmetin, apigenin and luteolin glycosides) from bergamot fruit juice [19,20,28]. BPF phyto-complex contains around 40% of flavonoids, but also sugars, salts and other natural compounds with a possible detoxifying activity [19,20]. A particular feature of certain flavonoid glycosides, abundant in bergamot juice and in BPF, such as bruteridin and melitidin, is the presence of covalently linked 3-hydroxy-3-methylglutaryl (HMG) moiety [28,29]. Computational studies have suggested that both molecules may bind to the catalytic site of HMG-CoA reductase and inhibit cholesterol synthesis by replacing the endogenous substrate HMG-CoA [29]. Such a theoretical mechanism has been proposed to explain BPF efficacy as a cholesterol-lowering food supplement in clinical trials performed on metabolic syndrome patients. These studies also demonstrated strong reduction of blood TGL levels and mild effects on glucose levels in individuals taking BPF [23,24,30]. These effects might depend on the ability of BPF flavonoids to stimulate AMPK, which should also explain proautophagic proprieties of these compounds in models of hepatic steatosis in vitro and in vivo [12,19,20]. Finally, BPF may also have anti-inflammatory activity, since bergamot juice extract have been shown to reduce inflammation in animal models of ischemia/reperfusion and inflammatory bowel disease [31,32].

In our previous work, we have validated an animal model of NAFLD, which displayed some characteristics of human NASH and have shown that the supplementation of BPF is an effective strategy to prevent NAFLD in CAF-fed rats [12]. Although prevention studies in animal models of NAFLD/NASH have significantly contributed to the understanding of this pathology, they have been less accurate in translating aspects of clinical trials. In fact, human trials address the efficacy of therapeutic interventions, in association with diet and life style modifications, which are the most effective ways to promote liver fat removal and amelioration of histological severity [1,14,33]. For this reason, the main goal of this this work was to analyze the effects of BPF supplemented to SC diet as the primary treatment on rats with advanced NAFLD/NASH. The second goal was to provide further characterization of pathological changes in livers from rats exposed to CAF diet for much longer times (26 weeks) than previously investigated (14 weeks) [12]. Although a normocaloric diet alone was sufficient to cause regression of steatosis, BPF improved its therapeutic effects, proving to be a potent anti-inflammatory and lipid-lowering food-supplement for the treatment of NAFLD/NASH, but not obesity.

## 2. Materials and Methods

### 2.1. Animal Procedures

Thirty-two male 5-week-old Rcc:Han WISTAR rats (Harlan Laboratories, Indianapolis, IN, USA) were housed individually in steel cages under controlled conditions (temperature 20 ± 2 °C, light 07:00–19:00). The animals had access to water and were fed ad libitum with standard chow (SC) diet 2016 (“SC”, energy value of 3.0 kcal/g, calories from protein 24%, calories from fat 18%, calories from carbohydrates 58%, Harlan Teklad) for 3 weeks before and during the experiment. This animal study was approved by a local animal welfare committee and by the Italian Ministry of Health, according to Legislative Decree 116/1992, which was in force when the study was proposed (before 4 March 2014).

### 2.2. Experimental Design

Starting from Week 8 of age, 24 rats were fed with CAF diet (see below) and 8 rats with SC diet for 15 weeks. After this period, 3 representative CAF and 3 SC rats were sacrificed for RNA isolation and NASH evaluation. At Week 16 of induction phase, 21 obese rats were evenly assigned to 3 experimental groups: CAF/CAF (7 rats), CAF/SC (7 rats) and CAF/SC+BPF (7 rats). CAF/CAF group continued to be fed with CAF diet, while CAF/SC and CAF/SC+BPF groups were switched to SC diet with and without BPF, respectively, for the subsequent 10 weeks. The remaining 5 lean rats continued to be fed with SC diet (SC/SC) for additional 11 weeks. During the intervention phase, CAF/SC+BPF rats were subjected to BPF treatment, which was supplemented to drinking water at suitable concentration to ensure an average 50 mg/kg/rat daily dosage. This dose was calculated as follows: the previously tested dose in humans, 1000 mg/100 kg = 10 mg/kg, was multiplied by 5 to account for higher metabolic rate in rodents, as previously described [12,34]. After 16 + 10 weeks of treatment, all animals were sacrificed for blood and tissue collection. 

### 2.3. Diet and Supplementation

The CAF feeding regimen contained 15% protein, 70% carbohydrates and 15% fat, in the form of cookies, milk chocolate snacks, crackers, cheese, processed meats, condensed milk, etc. [11,12]. It was provided in excess, together with normal SC diet. Caloric intake in CAF-fed rats was typically 110 kcal/day/rat, while SC diet provided 60 kcal/day/rat [12]. Nutritional composition of CAF diet components is provided in Table S1 in the Supplementary Materials.

BPF was prepared by the absorption on polystyrene resin columns and alkaline elution as described in detail in the European patent (No. EP 2 364 158 B1) and characterized for polyphenol content by high-pressure liquid chromatography [12,18,20]. It was kindly provided by Herbal and Antioxidant Derivatives S.r.l. (Bianco, RC, Italy). BPF diluted in drinking water was provided daily or every 2 days and during this time it remained stable, as it did not change color and taste. The amount of water and BPF was monitored to calculate the daily intake of BPF. The concentration of BPF added to drinking water varied from 1 to 2 mg/mL and was adjusted to rat body mass and daily water consumption to ensure a mean 50 mg/kg/rat/day dose over a 10-week period.

### 2.4. Blood and Tissue Collection

After the intervention phase, food was removed for 4 h before sacrifice under Zoletil 80 mg/kg and Dormitor anesthesia for blood and tissue collection. The blood was collected by cardiac puncture with heparinized 21G needle and divided for plasma (2 mL) and serum preparations (3–4 mL) in appropriate blood collection tubes. For blood tests on Week 4 of experiment, orbital sinus blood sampling was performed by an experienced veterinarian after 5% isoflurane anesthesia (Merial, Toluse, France), as described previously [12]. Before tissue collection, animals were perfused with 150 mM NaCl solution to remove blood and then collected. All the organs were weighed and divided for histological processing and shock frozen in liquid nitrogen for biochemical analysis.

### 2.5. Blood Analysis

Hematochemical parameters, such as TGL and glucose, were determined in the serum. The analyses were performed using commercial reagents on a Dimension EXL analyzer (Siemens Healthcare Diagnostics s.r.l., Milan, Italy). Routine blood counts were performed on EDTA-treated samples on Advia 2120 blood cell counter (Siemens).

The concentrations of insulin and leptin were measured using ELISA kits (Rat/Mouse Insulin ELISA Kit; Mouse Leptin ELISA Kit, EMD Millipore Corporation, Darmstadt, Germany) according to the manufacturers’ instructions. Approximate insulin resistance (IR) was calculated using the homeostasis model assessment (HOMA)-IR using the following formula: (glucose (mmol/L) × insulin (μU/mL))/22.5 [35].

The levels of alanine aminotransferase (ALT), aspartate aminotransferase (AST) and lactate dehydrogenase (LDH) were determined using commercial kits (Siemens Healthcare Diagnostics s.r.l., Milan, Italy) and automated biochemistry analyzer (Dimension EXL, Siemens Healthcare Diagnostics s.r.l.).

### 2.6. Histochemistry and Digital Image Analysis of LDs in Rat Liver Sections

The procedure for preparing liver tissue for cryosectioning and analysis of LDs was described previously [12]. Briefly, 10-µm liver sections were obtained by sectioning, using a cryostat at −20 °C (Leica Biosystems Inc., Buffalo Grove, IL, USA), and then histochemistry of LDs in perfused rat liver sections was performed by Oil Red O (ORO, Sigma-Aldrich, St. Louis, MO, USA) staining according to the previously described procedure [12].

Equivalent ORO plus hematoxylin-stained liver sections of each experimental group were first examined in bright-field with Leica Microscope DM4000B (Leica Microsystems GmbH, Wetzlar, Germany) equipped with 10×, 40× and 100× objective lenses. For each liver section, at least three independent images, from equivalent central lobe areas were captured at 10×, 40× and 100× magnification. For quantitative and qualitative LDs analysis, images of ORO-stained sections were processed and analyzed using a semi-automatic procedure implemented in Image J2x software package (National Institute of Health, Bethesda, MD, USA) and analyzed for percentage of total LDs area and the number of LDs.

### 2.7. Total Liver Lipid Assay

Total liver lipids were extracted according to the modified procedure of Folch et al. [12]. Briefly, frozen liver tissue (~400 mg) was thoroughly homogenized with 800 µL of deionized water (15 strokes). Four milliliters of chloroform/methanol (2:1 vol/vol) mixture (Sigma-Aldrich) was then added to the mix to generate a distinct organic and aqueous phase and extract lipids according to the previously described procedure [12].

### 2.8. Histology and Histological Examination

#### 2.8.1. Toluidine Blue Staining

Liver were quickly excised cut into small pieces, and fixed by direct immersion in 4% glutaraldehyde in phosphate-buffered saline (PBS 0.1 M, pH 7.2) for 48 h at 4 °C. After post-fixation for 2 h with osmium tetroxide (1% in the same buffer), samples were dehydration in an increasing series of ethanol and then embedded in epoxy resin (Araldite 502/Embed 812, Electron Microscopy Sciences, Hatfield, PA, USA). Semi-thin sections (1 µm) were stained with toluidine blue, observed and photographed by a LM LEITZ Dialux EB 20 (Leica Microsystems, Wetzlar, Germany) equipped with a digital camera.

#### 2.8.2. Silver Impregnation (SI)

Silver staining (SI) detects collagen III and B (argyrophilic reticulin) fibers, which are highly increased in liver fibrotic tissue [36]. A standard histochemical protocol of silver impregnation (SI) kit (Bio-Optica s.r.l., Milan, Italy) was applied, according to manufacturer guidelines. In brief, 4 µm-thick serial sections were obtained from a representative block of formalin-fixed, paraffin-embedded tissue, mounted on coated glass slides, and heated at 60 °C for 60 min. Hematoxylin/eosin (HE) staining was performed on the first section to observe the tissue morphology. The second serial section was used for the SI staining.

#### 2.8.3. Morphological Evaluation

Double-blind evaluation of all sections stained with TB, SI and HE as well as NAFLD activity scoring (NAS) were performed by two expert pathologists independently. NAS was performed on HE and TB stained sections for steatosis (score 0–3), lobular inflammation (score 0–3) and hepatocellular ballooning (score 0–2), using the NASH Clinical Research Network (CRN) scoring system as described previously [37]. Five animals (n = 3 sections for each animal) were scored for every experimental group.

### 2.9. Analysis of Cytokine Gene Expression

Small pieces (0.4–1 g) from the central part of the main lobe of rat livers were shock-frozen in liquid nitrogen and stored until needed at −80 °C. This choice of tissue portion was intended to minimize possible differences due to tissue heterogeneity within the lobe. Frozen tissue was fragmented and 50–100 mg samples were homogenized with glass douncer on ice with 1 mL of TRIzol Reagent (Life Sciences, Invitrogen, Carlsbad, CA, USA). Total RNA (totRNA) was extracted using the TRIzol reagent method followed by DNase treatment (Qiagen, Germantown, MD, USA). Total RNA was carefully quantified and its integrity was verified on 0.8% denaturing agarose gel. The expression of cytokines was analyzed by RT^2^ Profiler PCR array plates (Qiagen, SABiosciences, Frederick, MD, USA) for rats before the treatment (15 weeks) or by a standard reverse transcription followed by quantitative PCR (RT-qPCR) based on SYBR Green detection for rats after 10-week diet treatment.

For array analysis, total RNAs from three representative rats of the same experimental group were pooled (3 × 5 µg) and cDNA was synthesized from 500 ng of pooled RNA with Qiagen One Step RT-PCR kit according to the instructions (Qiagen). The relative gene expression was assayed by using PARN-157Z and PARN-084Z RT^2^ Profiler PCR arrays (SABiosciences) and, for the purpose of this article, only cytokine and housekeeping (HK) genes data were used. qPCR was performed on an iQ5 Real Time PCR (BioRad Lab., Hercules, CA, USA) using SYBR Green (cycling conditions: 95 °C, 10 min; 95 °C, 15 s; and 60 °C, 1 min; repeated for 40 cycles) and subsequent analyses were carried out according to the manufacturer’s recommended protocol. Each pooled cDNA was analyzed on four independent plates. The data from each CAF plate were compared to four SC plates. The data analysis was performed with dedicated software available at the GeneGlobe Data Analysis Center (http://www.qiagen.com/it/shop/genes-and-pathways/data-analysis-center-overview-page/). The same normalization method was used for all analyzed array plates i.e., manually selected HK genes. LDH was excluded from 6 standard HK genes.

For standard RT-qPCR analysis, totRNA was isolated as described above from 5–6 rats for each experimental group and RNA representing one rat was processed separately. cDNA was synthesized from 5 µg of totRNA with TransScript^®^ II First-Strand cDNA Synthesis SuperMix (Transbionovo, Carlo Erba Reagents, Cornareado (MI), Italy, cat. FC40AH30102) according to manufacturer instructions. RT-qPCR was performed on QuantStudio 3 Real Time PCR Detection System (Applied Biosystems Europe, Monza (MI), Italy), using TransStart Top Green qPCR SuperMix (Carlo Erba, cat. AQ131-01) with addition of a copy of rat cytokine-specific primers. These primers were previously tested for good performance in SYBR green-based detection of very low gene expression of liver cytokines [38] (see Table 1). The applied cycling conditions were: 95 °C, 10 min; 95 °C, 15 s; and 61 °C, 1 min; repeated for 40 cycles. Samples were analyzed in triplicate with hypoxanthine phosphoribosyl transferase 1 (Hrpt1) as a HK control. Only results with the amplification of a single product, as verified by melting curve analysis, were considered. Relative gene expression was calculated according to the 2^−ΔΔCT^ method using the SC liver tissue cDNA (15 weeks on SC diet) as a starting point control.

### 2.10. Data Analysis and Statistical Procedures

Animal experiment was performed one time and the final mean values were based on at least 5 animals for each experimental group, except for leptin Elisa assay and for mRNA profiling experiment, where RNA pools from 3 rats were assayed. The data for one animal were a mean of at least three triplicate measurements (except for blood test) with standard deviation (SD). For statistical analysis, Prism 7.0 GraphPad software (GraphPad Inc., San Diego, CA, USA) was used or Excel Office 365. Nonparametric Mann-Whitney U test (comparisons between groups with no assumption about the scatter of the data) was performed for majority of datasets. Otherwise, the statistical method is indicated in the figure legend.

## 3. Results

### 3.1. Supplementation of SC Diet with BPF Has a Powerful Effect on Levels of Blood TGL

**Figure 1:**
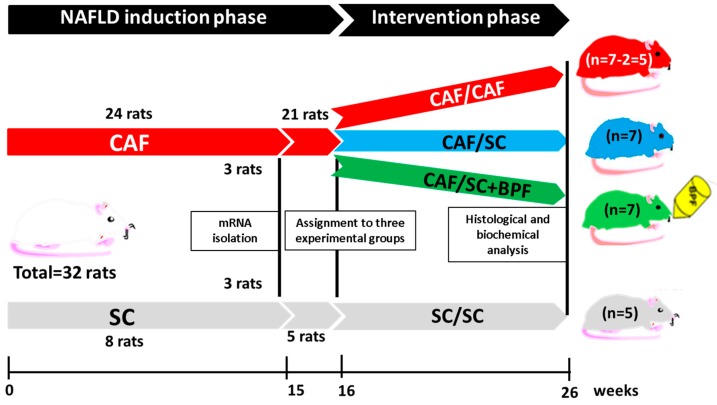
The experimental design and timeline of the study presented in this work: NAFLD/NASH induction phase for 16 weeks plus the intervention phase for 10 weeks. Black lines and boxes indicate the time of sample collection.

**Figure 2:**
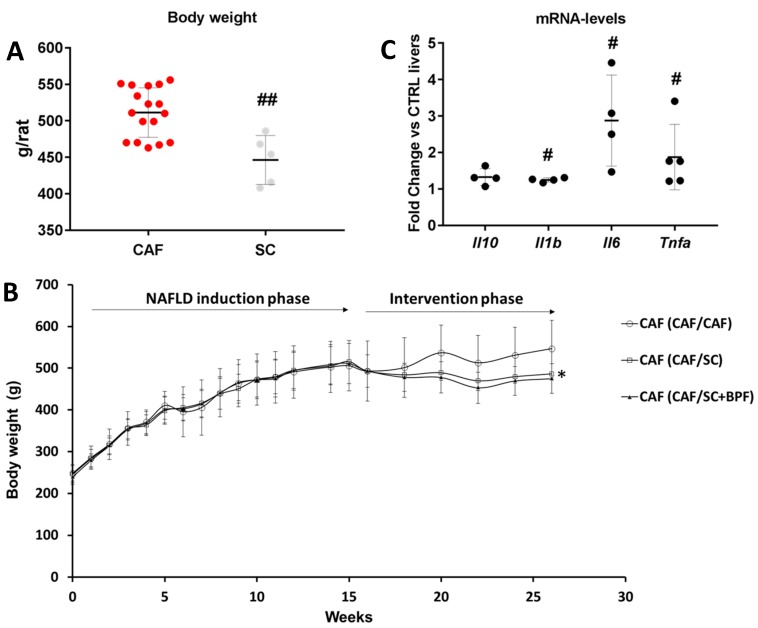
Starting conditions after the induction phase (16 weeks of CAF diet). (**A**) Distribution of body weight in rats fed with CAF diet (n = 21) or control SC diet (n = 5) after NAFLD induction phase (16 weeks). ## Statistically significant difference at *p* ˂ 0.003 when compared to SC control group. (**B**) Body weight changes over the entire period of the study. Data are represented as means ± SD (n = 7), * *p* < 0.05 indicates significant differences between CAF/CAF and CAF/SC groups. (**C**) Significant upregulation of the gene expression of inflammatory cytokines in rat livers at week 15 of CAF diet, before the intervention phase with SC diet. The graph shows the results of cytokine-related mRNA expression profiling performed on 96-well RT^2^ PCR array plates in three pooled RNA liver samples from three rats. The fold change of relative mRNA levels in CAF livers compared SC and normalized to five HK genes, is presented. Each dot shows the fold change value from one RT^2^ array plate (three CAF livers vs. SC livers), and the line shows the mean of presented data. Statistical significance at *p* ≤ 0.02 (#), when compared with control SC livers is reported according to GeneGlobe software (Qiagen), which calculates the *p* values based on a Student’s *t*-test of the replicate 2^-ΔΔCT^ values for each gene in the control and treatment groups.

To assess the effect of BPF supplementation to the low-sugar/fat diet regime as a therapeutic treatment for advanced NAFLD/NASH, we induced NAFLD with features of NASH in 21 Wistar male rats by feeding them with CAF diet for 16 weeks (induction phase), exactly as described previously [12]. These animals were then divided into three groups for the intervention phase of the experiment. 14 animals were subjected to SC diet with (CAF/SC+BPF) and without BPF (CAF/SC), while 7 rats continued with CAF diet (CAF/CAF) for further 10 weeks, according to the scheme in Figure 1. A small group of 5 rats was maintained on normocaloric SC diet during induction and intervention phases of the experiment as a basic control group (SC/SC) (Figure 1). After the induction phase, there was a statistical difference in the body weight between SC control and CAF animals (Figure 2A). As expected, the diet change during the intervention phase led to a significant reduction of the mean body weight in CAF/SC rats as compared to CAF/CAF group (Figure 2B). No significant effects of BPF on the body weight were observed in animals fed SC diet and consuming bergamot polyphenols with respect to CAF/SC rats.

Furthermore, CAF/CAF rats had developed severe NAFLD, characterized by a massive accumulation of LD lipid droplets and infiltration of Kupffer cells, according to previous observations in livers exposed to CAF diet for 14 weeks [12], indicating a histological severity compatible with NASH. To evaluate the presence of NASH, we performed gene expression profiling of pro- and anti-inflammatory cytokines, by RT-qPCR in 15-week CAF-fed rat livers. The results indicated a clear up-regulation of proinflammatory cytokines in CAF-fed livers when compared to control (SC) livers at Week 15. In particular, we observed a statistically significant increase of Interleukin 1β (*Il1b*), Interleukin 6 (*Il6*) and Tumor necrosis factor α (*Tnfa*) and no significant change in Interleukin 10 (*Il10*) gene expression (Figure 2C). These experiments yielded further information on Interferon gamma (*Ifng*) and Transforming growth factor beta (*Tgfb*) expression in 15-week CAF livers, but it was below a detection limit in case of Ifnγ or unchanged with respect to 15-week control livers in case of *Tgfb* (data not shown). Taking together, these data indicate CAF diet in Wistar rats induces NASH.

**Figure 3:**
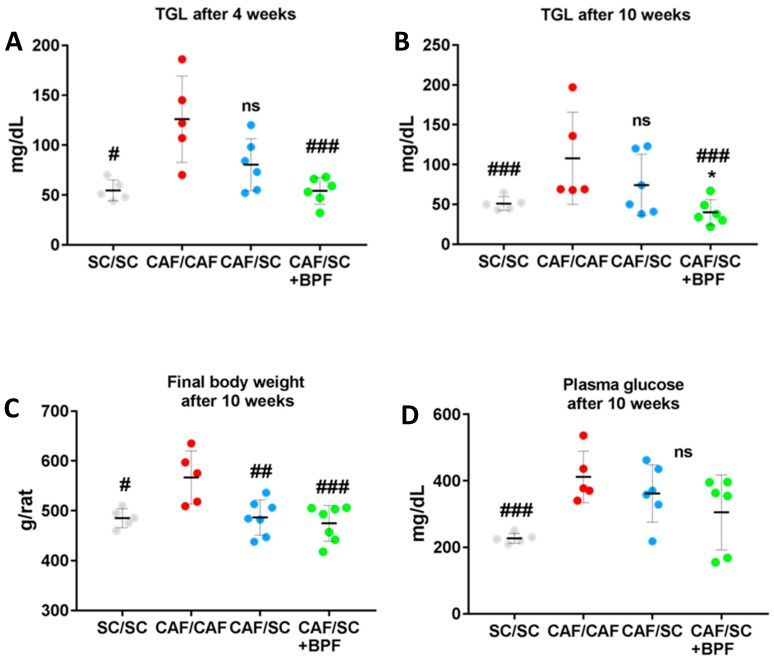
Supplementation of SC diet with BPF causes a reduction of blood TGL, but BPF effects on blood glucose and body weight loss are not significant. Serum TGL (**A**,**B**) and glucose (**D**) were measured after 4–5 h fasting in CAF rats treated for 4 or 10 weeks with SC diet ± BPF. #, ###, statistically significant difference vs. CAF/CAF group at *p* ≤ 0.05 and *p* ≤ 0.006, respectively. *, CAF/SC vs. CAF/SC+BPF at *p* ≤ 0.5. (**C**) Distribution of final body weight after 10 weeks of the intervention phase. ##, ###, statistically significant difference vs. CAF/CAF at *p* ≤ 0.02 and 0.002, respectively.

We observed a significant decrease of final body weight in CAF/SC and CAF/SC+BPF groups compared to CAF/CAF group, but BPF treatment did not cause any further reduction of body weight compared to CAF/SC group (Figure 3A). Among the CAF/CAF group rats, one died prematurely due to heart attack and another one could not feed due to a dentition defect, so it was excluded from statistical analysis.

Next, we tested whether the switch to SC diet and BPF treatment reduced blood parameters which are elevated in CAF/CAF diet-induced metabolic syndrome. We observed that triglyceridemia was reduced in CAF/SC rats after both 4 and 10 weeks of SC diet (Figure 3A,B). However, the concomitant administration of BPF resulted in a further reduction of blood TGL with significant differences between CAF/SC and CAF/SC+BPF groups at Week 10. Importantly, no significant differences in body weight were observed between CAF/SC and CAF/SC+BPF and SC/SC groups at Week 10 (Figure 3C). Similarly, no significant differences in blood glucose were found between the same experimental groups (Figure 3D).

These data suggest that supplementation of CAF/SC diet with BPF has strong effects on blood TGL, but modest effects on obesity and diabetes.

### 3.2. BPF Augments LDs Loss and Reduces Hepatic Inflammation When Supplemented to SC Diet during Recovery from NASH

**Figure 4:**
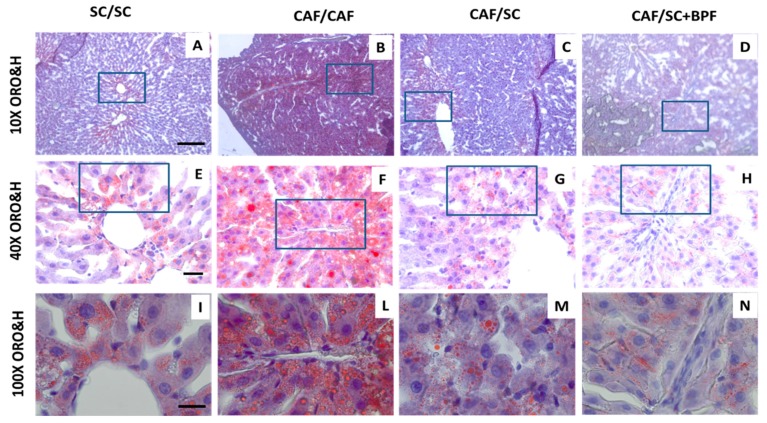
Strong LDs reduction in CAF/CAF livers after 10 weeks of treatment with standard chow (CAF/SC) diet or SC diet supplemented with BPF (CAF/SC+BPF). ORO and Hematoxylin (ORO&H) staining of representative liver sections from four treatment groups: SC/SC (26 weeks) (**A**,**E**,**I**); CAF/CAF (16 + 10 weeks) (**B**,**F**,**L**); CAF/SC (**C**,**G**,**M**); and CAF/SC+BPF diet (**D**,**H**,**N**). ORO&H staining allows visualization of unsaturated fatty acids and neutral fats (red, ORO) and nuclei (blue stained). Scale bars are as follows: (A–D) 200 μm ; (E–H) 40 μm; and (I–N) 20 μm. Boxes indicate magnified regions. 10×, 40× and 100× indicate objective magnifications.

To address the effect of SC diet and BPF treatment on CAF diet-induced fat accumulation, serial sections of livers from each diet group were evaluated using ORO histochemistry. Hematoxylin and ORO staining of liver sections showed accumulation of numerous, big and giant LDs in hepatocytes of CAF-fed rats (Figure 4B,F,L). This phenotype was greatly attenuated in CAF/SC (Figure 4C,G,M) and CAF/SC+BPF (Figure 4D,H,N) groups.

**Figure 5:**
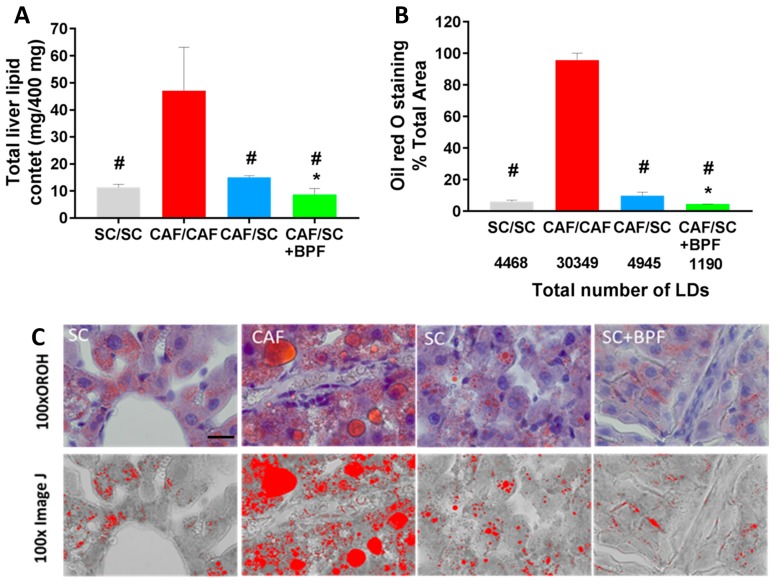
BPF supplementation to SC diet causes stronger reduction of total liver lipid content, size and numbers of LDs compared to SC diet alone for 10 weeks. (**A**) Total lipids were extracted from 400 mg of liver tissue by Folch method and gravimetrically determined. (**B**) Quantification of the total area occupied by LDs in ORO-stained sections revealed a significant increase in CAF/CAF vs. other groups (# *p* ≤ 0.02), while BPF reduced the extent of intrahepatic fat accumulation, when compared to normocaloric diet (* *p* ≤ 0.02 vs. CAF/SC). Each bar represents the median of four animals ± SD. (**C**) Binary transformation of ORO staining using ImageJ. 100x, objective magnification; scale bar = 20 μm.

The above findings were confirmed by total lipid content analysis (Figure 5A). A significant reduction in total lipids was observed in CAF/SC livers compared to CAF/CAF livers. Furthermore, the supplementation of SC diet with BPF resulted in a substantial reduction in total lipids content in CAF/SC+BPF group compared to CAF/SC group.

Next, we evaluated morphometric parameters of ORO-stained LDs in liver sections from the four experimental groups (Figure 5B,C). In livers from CAF/CAF rats, LDs covered a big area of the digitalized image, whereas a relatively small area was occupied by LDs in livers from SC/SC, CAF/SC and CAF/SC+BPF rats, respectively (Figure 5B,C). Moreover, the total number of LDs was reduced by six-fold in CAF/SC livers compared with CAF/CAF livers. A significant difference in LDs numbers between the BPF-treated CAF/SC and CAF/SC groups was also reported (Figure 5B).

**Figure 6:**
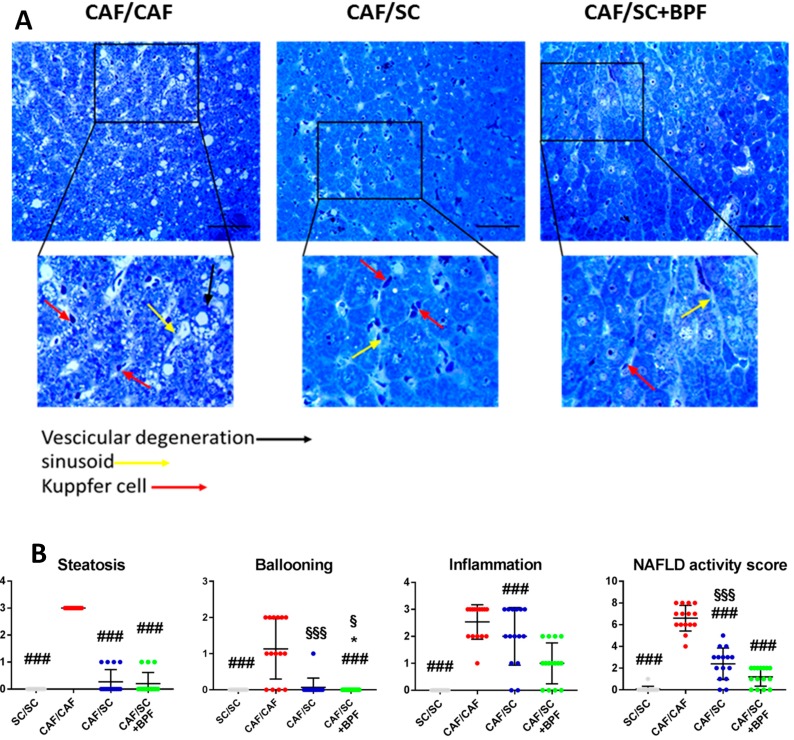
Histological evaluation of NASH. (**A**) Toluidine-blue staining of representative liver sections from three different treatment groups CAF/CAF (16 + 10 weeks), CAF/SC, and CAF/SC+BPF diet. Scale bar 50 μm. (**B**) Quantification of NAFLD Activity Score (NAS) parameters such as steatosis, ballooning and inflammation. Data are represented as means ± SD (n = 15 (five animals and three independent sections for each animal)). * *p* ≤ 0.01 denotes differences statistically significant CAF/SC vs. CAF/SC+BPF; ### *p* ≤ 0.0007 CAF/CAF vs. SC/SC, CAF/SC, CAF/SC+BPF; § *p* ≤ 0.01 SC/SC vs. CAF/SC+BPF; §§§ *p* ≤ 0.01 SC/SC vs. CAF/SC. For statistical analysis, Kruskal-Wallis test was performed.

Moreover, different sections of livers from each diet group were also evaluated using toluidine-blue staining. Microscopic examination of toluidine-stained semi-thin sections of resin-embedded livers from CAF/CAF, CAF/SC and CAF/SC+BPF rats revealed important changes in CAF/SC and CAF/SC+BPF liver histology with respect to CAF/CAF group (Figure 6 and Figure S1). CAF/SC and CAF/SC+BPF liver parenchyma appeared homogeneous with a regular distribution of hepatocytes. The hepatocytes showed uniform size with large rounded nuclei usually located in the center of the cells and cytoplasmic glycogen granules, but no LDs. In contrast, CAF/CAF livers presented large areas of steatotic tissue with a completely disorganized parenchyma lacking the natural arrangement of hepatocytes. The cytoplasm of hepatocytes appeared highly vacuolated and with a large amount of glycogen granules and LDs. Ballooning hepatocytes, considered a histological hallmark of NASH [39], were also found, more frequently at the portal region (Figure 6A and Figure S1).

Moreover, the sinusoid organization was irregular in CAF/CAF livers compared to CAF/SC and CAF/SC+BPF livers. Within the lumen of sinusoids, large populations of Kupffer cells were observed and some more rounded lymphocytes. Importantly, Kupffer cells and lymphocytes were also present in livers recovering from NASH, but fewer inflammatory cells were observed in CAF/SC+BPF group when compared to SC group (Figure 6B and Figure S1). Quantification of lobular inflammatory foci, by NAS scoring (Figure 6A,B), further confirmed that BPF supplementation has important anti-inflammatory effects. To detect fibrosis, we evaluated reticular fibers, which are thick and form bundles in connective tissue. Reticular fibers in normal liver tissue are thin and less abundant. They are usually not visible in histological sections stained with hematoxylin/eosin or toluidine blue, but can be demonstrated by using silver impregnation (SI). SI staining revealed many regions of thick fibers networks, mainly in the proximity of blood vessels in CAF/CAF liver samples. On the contrary, fewer and only thin reticular fibers were present in CAF/SC and CAF/SC BPF livers.

### 3.3. Analysis of Plasmatic Levels of Insulin and Leptin in SC/SC, CAF/CAF, CAF/SC and CAF/SC+BPF Diet Fed Rats

The dysregulation of lipid metabolism in NAFLD can have devastating consequences on glucose homeostasis and lead to insulin resistance (IR) and type 2 diabetes. On the other hand, IR plays a primary role in triggering a series of reactions that lead to hepatic steatosis [40].

**Figure 7:**
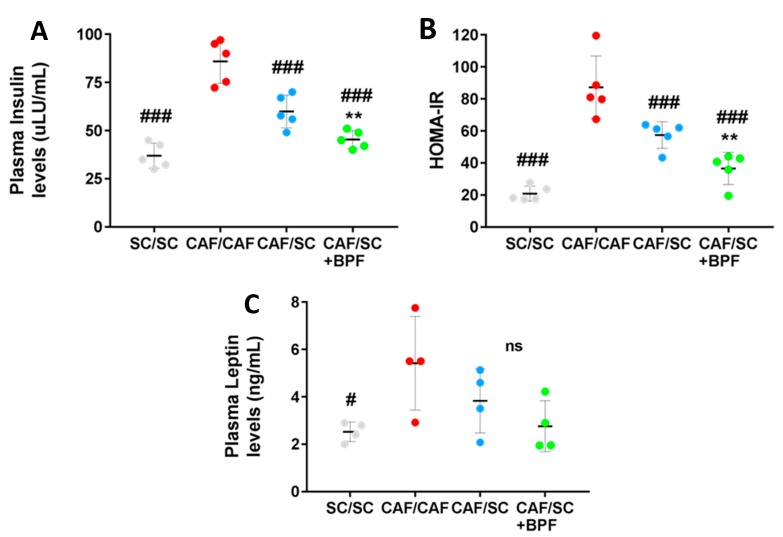
Fasting plasma levels of insulin and leptin and insulin resistance after treatment with SC diet ± BPF for 10 weeks. (**A**) Insulin; and (**C**) leptin were assessed by ELISA in blood samples collected at the end of treatments after 4–5 h fasting. Insulin and leptin levels are elevated in CAF-fed rats when compared to SC/SC ((### *p* ≤ 0.008 and # *p* ≤ 0.02, insulin and leptin, respectively, CAF/CAF vs. SC/SC). (**B**) Insulin sensitivity was measured by HOMA-IR index (** *p* ≤ 0.01 CAF/SC+BPF vs. CAF/SC). (**A**–**C**) Each horizontal line and vertical bar represent the median ± SD, respectively, of n = 4–5 rats.

To test whether BPF supplementation in association with healthy diet could improve insulin sensitivity, we analyzed plasma levels of insulin in the four experimental groups. As expected, CAF/CAF rats showed much higher insulin levels than SC/SC rats (Figure 7A), suggesting IR.

Importantly, the fasting plasma insulin concentrations of the rats fed with CAF/SC diet supplemented with BPF were significantly lower than those of the rats fed with CAF/SC diet (Figure 7A). In addition, the HOMA-IR index was also significantly lower in CAF/SC+BPF group when compared with CAF/SC group (Figure 7B). HOMA-IR index has been shown to have a strong and direct correlation with the insulin tolerance tests in Wistar rats, and can be used as a surrogate marker of IR in rats [35,41,42]. Thus, BPF supplementation significantly improved the effect of the diet on HOMA-IR values.

The IR is often strongly associated with leptin-resistance in obese subjects [26]. Leptin is a protein hormone secreted by adipose cells that plays a role in helping the body to balance food intake with energy expenditure. The serum leptin levels were significantly elevated in rats fed with CAF/CAF diet compared with those fed with the SC/SC diet; however, CAF/SC diet, along BPF supplementation, did not cause any further significant decrease in serum leptin levels (Figure 7C).

### 3.4. Anti-Inflammatory Effect of BPF in CAF/SC Diet Treated Livers

Following histological analysis, we evaluated liver injury parameters such as ALT, AST and LDH. However, ALT and AST values were not significantly altered in CAF/CAF group with respect to SC/SC group (Supplementary Materials, Figure S2A,B), suggesting that these biochemical tests do not correlate well with liver inflammation in our rat NASH model. Conversely, we found that measuring LDH level might be helpful, as it is significantly elevated in CAF/CAF group compared to all other groups. In addition, we found that BPF supplementation ameliorated the LDH values in the diet intervention groups (Supplementary Materials, Figure S2C).

**Figure 8:**
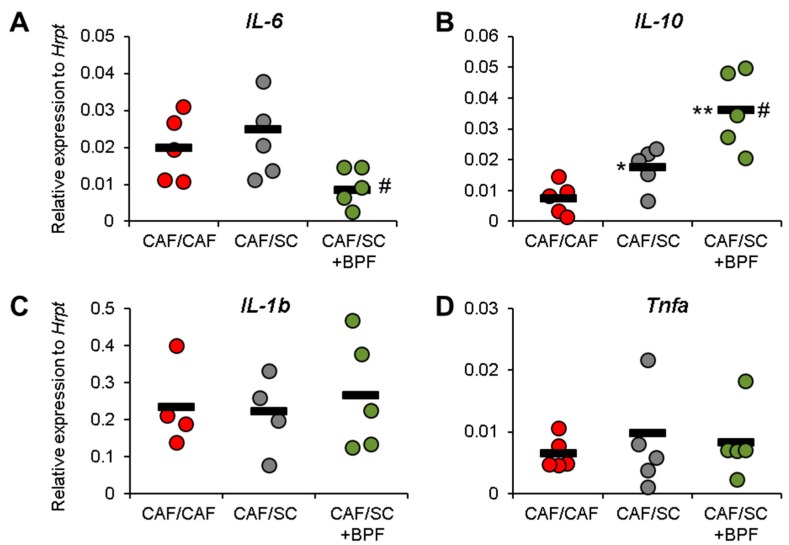
Effect on pro-inflammatory cytokines and anti-inflammatory *Il-10* upon BPF supplementation to CAF/SC diet in rat livers after the intervention phase (10 weeks). RT-qPCR analysis of mRNA levels of inflammatory cytokines *Il1b*, *Il6*, and *Tnfa* and anti-inflammatory cytokine *Il10*. The graphs show relative gene expression versus HK gene *Hrpt.* Each dot represents the average relative expression for one rat analyzed at least in triplicate by standard methods, and lines are the mean values of presented dots. Statistical analysis was performed by unpaired, two-tailed *t* test. *, #, Statistically significant difference at *p* < 0.05 when compared with CAF/CAF rats or when compared with CAF/SC rats, respectively. **, Statistically significant difference as above at *p* < 0.01.

Finally, we addressed if inflammatory parameters could be improved by SC diet and BPF supplementation. RT-qPCR analysis indicated that, although the CAF/SC diet did not affect gene expression levels of pro-inflammatory cytokines 1β, *Il-6* and *Tnfa* (Figure 8A,C,D), BPF treatment resulted in a significant reduction in the mRNA levels of *Il-6* (Figure 8A).

Furthermore, the mRNA levels of anti-inflammatory cytokines *Il-10* were significantly elevated in rats fed with the CAF/SC compared with those fed with the CAF/CAF diet and BPF supplementation caused a further increase of mRNA levels of *Il-10* (Figure 8B) suggesting that BPF supplementation exerts anti-inflammatory effects on steatohepatitis.

## 4. Discussion

Our previous work showed that the supplementation of hypercaloric diet with an extract of natural *Citrus* polyphenols from bergamot (BPF) prevents NAFLD through the stimulation of autophagy in the liver [12], which was further confirmed by in vitro studies [19,20]. The main aim of the present study was to examine the effect of BPF supplementation to normocaloric diet on CAF diet-induced advanced NAFLD in therapeutic regime. Most of the studies on animal models of NAFLD or NASH, including our previous work, address the preventive effect of a treatment [43,44,45,46,47]. Conversely, in this paper, we used a therapeutic approach which is more appropriate for translational interpretation of the results, since it has been designed as a typical clinical study on sick patients, in which a dietary intervention is associated or not with a pharmacological or nutraceutical treatment [4,5,15].

As expected, 10-week SC diet intervention phase caused weight loss (~15%) associated with a dramatic decrease of total liver lipid content (~70%) and ORO staining (~85%). Furthermore, we observed a powerful effect of SC diet on total liver lipid content, number and size of LDs. Indeed, in our model, abundant and large LDs accumulated in the cytoplasm of hepatocytes of rats fed with CAF diet, occupying an area about 10-fold greater than that in SC-fed group. Upon administration of normocaloric SC diet, the LDs total area as well as LDs total number were significantly reduced (Figure 5A–C). Importantly, the concomitant administration of BPF during the intervention phase significantly restrained liver fat content and number and size of LDs with respect to SC group. 

In line with this observation, BPF had important effects on blood TGL, leading to a significant reduction of TGL compared to CAF/SC group, after only four weeks of BPF supplementation to SC diet. On the other hand, BPF effects on blood glucose were modest with respect to CAF/SC group even after 10 weeks, although they could likely become statistically significant, if the tested groups were bigger.

Similarly, BPF did not affect body weight, but we cannot exclude a weak effect demonstrable by increasing the number of tested rats. Human NAFLD is very sensitive to the diet and an intensive lifestyle intervention focused on diet with a goal of 7–10% weight reduction leads to significant improvement in liver histology in patients with NASH [33,48]. Weight loss improves steatosis, reduces hepatic inflammation and hepatocellular injury, including fibrosis, suggesting that body weight and liver fat accumulation are strictly correlated [1]. However, our data indicate that liver and body fat might be regulated by distinct mechanisms. It seems that liver fat accumulation is sensitive to pleiotropic effects of flavonoids, while body fat is less affected. Our observations are in line with a recent study, that shows a potent effect of Polyphenol-Rich Rutgers Scarlet Lettuce extract on NAFLD induced with high-fat diet and treated with low-fat diet and no effect on body weight [49]. In humans, isocaloric diet modifications also improve NAFLD, but has little effect on body weight [14]. In contrast, most of the studies in the literature performed using different polyphenol-rich extracts as a preventive measure in animal models of NAFLD, show concomitant reduction of body weight gain and liver lipid content [43,47,50,51,52]. This difference arises probably from the fact that other nutraceutical approaches are less liver-specific compared to BPF and they are assayed in NAFLD prevention studies.

Our data regarding leptin modulation support this hypothesis. In fact, BPF improved insulin sensitivity compared to SC diet treatment alone, but had no additional effect on leptin levels (Figure 7A,C). CAF rats as well as being obese showed higher plasma levels of insulin and leptin. Leptin is an adipokine that is primarily secreted from adipose tissue and has a critical role in the regulation of body weight and fat mass. Circulating leptin is strongly associated with both subcutaneous and visceral fat, and different studies demonstrate that obesity might induce a state of leptin resistance, when, despite high plasma levels of leptin, the biological activity of this hormone is very low. Several studies indicate that leptin expression is stimulated by insulin [53,54], whereas other studies suggest that high leptin concentrations may contribute to IR [2,55]. Importantly, we found that, although BPF supplementation improved insulin resistance determining a significant decrease in insulin levels and HOMA-IR index, it had no significant effects on body fat and leptin levels. These results suggest a specific action of BPF on liver fat accumulation and glucose metabolism rather than on body weight loss.

The most important finding of this paper is that BPF shows a significant anti-inflammatory effect on NASH livers, with respect to 10-week SC diet treatment, suggesting that SC diet alone has limited effects on inflammation at this timepoint, while BPF supplementation accelerates recovery during the intervention phase. CAF-treated livers were characterized by the presence of ballooning hepatocytes and increased numbers of Kupffer cells and some lobular inflammatory loci. This correlated with increased expression of all pro-inflammatory cytokines in CAF livers, with respect to SC livers, that could be detected already after 15 weeks of CAF-induction phase. In particular, we observed strongly increased levels of *Tnfα* and *Il6* mRNAs and moderately increased *Il1b* gene expression (Figure 2C), which are the main pro-inflammatory cytokines typically elevated in NASH. This was associated with a moderate increase of anti-inflammatory *Il10* mRNA, likely as a compensatory mechanism. Interestingly, 10-week SC diet intervention did not lead to significant changes in pro-inflammatory cytokine gene expression in CAF/SC livers (Figure 8), even though we found much less steatosis and improved NAS with respect to CAF/CAF livers. This may be related to still ongoing remodeling and recovery process in CAF/SC livers at this time point. However, low gene expression levels as well as an intrinsic expression variability of cytokines in liver samples, makes it difficult to detect subtle changes. This might be true for *Tnfa* and *Il6*, but not for *Il1b*, which has relatively high hepatic expression and we detected no significant differences between groups. However, we could show that BPF flavonoids induced suppression of pro-inflammatory *Il6* and potently boosted the gene expression of anti-inflammatory *Il10*, which was moderately up-regulated also in CAF/SC livers (Figure 8). In addition, we did not observe detectable changes in other cytokines in CAF/SC+BPF livers with respect to CAF/CAF and CAF/SC livers. This suggests that, during diet-induced recovery from NASH, some inflammatory features persist, while BPF reduces inflammation by acting on *Il6* and *Il10*. In fact, we found increased numbers of immune effector cells, mainly Kupffer and some lymphocytes, infiltrating CAF/SC livers while much fewer immune cells in CAF/SC+BPF at week 10 of dietary intervention supplemented with bergamot polyphenols. To our knowledge, this is the first time that some persistent inflammation is described in animal models of NASH after dietary intervention, despite a prominent reduction in LDs. This may be explained by still ongoing phagocytosis of apoptotic cells and debris mediated by Kupffer cells, accompanying hepatic tissue regeneration and healing. Considering that CAF diet livers were badly compromised by 16-week CAF induction phase, it is likely that 10-week SC-intervention phase is not sufficient to obtain a full regression of NASH. We propose that this recovery process is strongly accelerated by antioxidant and anti-inflammatory supplements such as BPF.

The anti-inflammatory activity of *Citrus* flavonoids is well-documented in the scientific literature and it has been studied both for flavanone and flavone glycosides as well as for their aglycones [56,57,58]. This is true for naringin and hesperidin [27], which are abundant in BPF as well as for diosmetin, apigenin, and luteolin glycosides, which are less abundant components of BPF [19,20]. The anti-inflammatory properties of neoeriocitrin, the most abundant flavanone-7-O-neohesperidoside in bergamot fruits and BPF, have not been formally demonstrated, because it has been poorly investigated being a rare flavonoid in other *Citrus* plants [18,28,30]. However, it been shown that its aglycone, eriodictyol, restrained the elevation of plasma IL-6 and C-reactive protein (hs-CRP) in mice fed high fat diet, suggesting that other eriodictyol glycosides, such as neoeriocitrin, are also active [59]. In fact, glycosides undergo deglycosylation by gut bacteria and usually are adsorbed by enterocytes as aglycones or their metabolites [18,27,60], therefore the in vivo studies on flavonoid aglycones and glycosides should generally yield overlapping results. In fact, the intestinal absorption of naringenin glycosides is comparable to the aglycone [60]. However, their bioavailability depends on the mode of administration and it is much higher, over 30% for naringenin-O-glucoside, if it is given to rats as a food supplement [60], as in our study. Considering high amount and relatively good bioavailability of naringenin, hesperetin and eriodictyol glycosides [61], it is likely that the main BPF flavanones are responsible for majority of immunomodulatory activity present in BPF. Future comparative studies addressing the anti-inflammatory efficacy of individual bergamot flavonoids should answer this question. Nevertheless, by analogy to the study on proautophagic activity of *Citrus* flavonoids [19,20], we expect that the combinatorial effect of the BPF phytocomplex may be quite different and likely superior to the effects of individual polyphenols.

The second goal of our study was to characterize rat livers after longer NAFLD/NASH induction with CAF diet. In our hands, Wistar rats feeding with CAF diet for 26 weeks induced, beside inflammation, different other NASH features, such as ballooning and portal fibrosis, documented here by SI staining of liver sections (Figure S3). This approach can be successfully used to visualize areas of liver fibrosis [62,63], as it detects collagen III and B fibers, which are highly increased in liver fibrotic tissue [36]. Thus, our data suggest that CAF diet is fibrogenic, even in Wistar rats, typically resistant to fibrosis in response to high-fat diet [64]. The presence or absence of fibrosis in response to CAF diet likely depends on the animal strain. In fact, CAF diet induced portal fibrosis in livers and severe fibrotic changes in heart and kidneys of BALB/c mice treated for 15 weeks [65], while fibrosis can be easily induced in certain mouse and rat strains even if the animals are treated with less effective high-fat diet [37]. To our knowledge, this the first report documenting the presence of liver fibrosis in CAF-diet treated rats.

## 5. Conclusions

For a long time, NAFLD has been underestimated as a condition of poor clinical relevance. However, the rising incidence of NASH in the Western world, also among children, suggests an urgent need to develop strategies of effective pharmacological or nutraceutical treatment.

In light of the promising evidence presented in this paper, the supplementation of BPF to normocaloric diet may represent a useful anti-inflammatory approach to accelerate patients recovery from advanced NAFLD/NASH.

## Figures and Tables

**Table 1:** The primers sequences used in RT-qPCR.

Gene		Sequences (5′-3′)
*Il1b*	Forward Reverse	CACCTCTCAAGCAGAGCACAG GGGTTCCATGGTGAAGTCAAC
*Il6*	Forward Reverse	TCCTACCCCAACTTCCAATGCTC TTGGATGGTCTTGGTCCTTAGCC
*Il10*	Forward Reverse	GTTGCCAAGCCTTGTCAGAAA TTTCTGGGCCATGGTTCTCT
*Tnfa*	Forward Reverse	AAATGGGCTCCCTCTCATCAGTTC TCTGCTTGGTGGTTTGCTACGAC
*Hrpt*	Forward Reverse	CTCATGGACTGATTATGGACAGGAC GCAGGTCAGCAAAGAACTTATAGCC

## Supplementary Materials

The following are available online at http://www.mdpi.com/2072-6643/10/11/1604/s1, Figure S1: Toluidine-blue staining of representative liver sections from 3 different treatment groups: CAF/CAF (16 + 10 weeks), CAF/SC, and CAF/SC+BPF, Figure S2: Evaluation of serum biochemical parameters of liver injury, Figure S3: Histological analysis of liver fibrosis in Wistar rats exposed to CAF diet for 26 weeks (CAF/CAF) or exposed to CAF diet for 16 weeks and then to SC or SC+BPF diet for 10 weeks, Table S1: List of the food items used to assemble *Cafeteria* (CAF) diet and related nutritional values.

## References

[1] Hannah W.N., Harrison S.A. (2016). Lifestyle and dietary interventions in the management of nonalcoholic fatty liver disease. Dig. Dis. Sci..

[2] Haslam D.W., James W.P. (2005). Obesity. Lancet.

[3] Mouzaki M., Allard J.P. (2012). The role of nutrients in the development, progression, and treatment of nonalcoholic fatty liver disease. J. Clin. Gastroenterol..

[4] Oseini A.M., Sanyal A.J. (2017). Therapies in non-alcoholic steatohepatitis (nash). Liver Int..

[5] Della Corte C., Carpino G., De Vito R., De Stefanis C., Alisi A., Cianfarani S., Overi D., Mosca A., Stronati L., Cucchiara S. (2016). Docosahexanoic acid plus vitamin d treatment improves features of nafld in children with serum vitamin d deficiency: Results from a single centre trial. PLoS ONE.

[6] Marzuillo P., Del Giudice E.M., Santoro N. (2014). Pediatric non-alcoholic fatty liver disease: New insights and future directions. World J. Hepatol..

[7] Wesolowski S.R., Kasmi K.C., Jonscher K.R., Friedman J.E. (2017). Developmental origins of nafld: A womb with a clue. Nat. Rev. Gastroenterol. Hepatol..

[8] Elmaogullari S., Demirel F., Hatipoglu N. (2017). Risk factors that affect metabolic health status in obese children. J. Pediatr. Endocrinol. Metab..

[9] Sampey B.P., Freemerman A.J., Zhang J., Kuan P.F., Galanko J.A., O’Connell T.M., Ilkayeva O.R., Muehlbauer M.J., Stevens R.D., Newgard C.B. (2012). Metabolomic profiling reveals mitochondrial-derived lipid biomarkers that drive obesity-associated inflammation. PLoS ONE.

[10] Gasparin F.R.S., Carreno F.O., Mewes J.M., Gilglioni E.H., Pagadigorria C.L.S., Natali M.R.M., Utsunomiya K.S., Constantin R.P., Ouchida A.T., Curti C. (2018). Sex differences in the development of hepatic steatosis in cafeteria diet-induced obesity in young mice. Biochim. Biophys. Acta. Mol. Basis Dis..

[11] Sampey B.P., Vanhoose A.M., Winfield H.M., Freemerman A.J., Muehlbauer M.J., Fueger P.T., Newgard C.B., Makowski L. (2011). Cafeteria diet is a robust model of human metabolic syndrome with liver and adipose inflammation: Comparison to high-fat diet. Obesity.

[12] Parafati M., Lascala A., Morittu V.M., Trimboli F., Rizzuto A., Brunelli E., Coscarelli F., Costa N., Britti D., Ehrlich J. (2015). Bergamot polyphenol fraction prevents nonalcoholic fatty liver disease via stimulation of lipophagy in cafeteria diet-induced rat model of metabolic syndrome. J. Nutr. Biochem..

[13] Takahashi Y., Sugimoto K., Inui H., Fukusato T. (2015). Current pharmacological therapies for nonalcoholic fatty liver disease/nonalcoholic steatohepatitis. World J. Gastroenterol..

[14] Della Pepa G., Vetrani C., Lombardi G., Bozzetto L., Annuzzi G., Rivellese A.A. (2017). Isocaloric dietary changes and non-alcoholic fatty liver disease in high cardiometabolic risk individuals. Nutrients.

[15] Dongiovanni P., Lanti C., Riso P., Valenti L. (2016). Nutritional therapy for nonalcoholic fatty liver disease. J. Nutr. Biochem..

[16] Abenavoli L., Milic N., Luzza F., Boccuto L., De Lorenzo A. (2017). Polyphenols treatment in patients with nonalcoholic fatty liver disease. J. Transl. Int. Med..

[17] Son C.G., Wei Z., Raghavendran H.B., Wang J.H., Janda E. (2017). Medicinal herbs and their active compounds for fatty liver diseases. Evid. Based Complement. Alternat. Med..

[18] Janda E., Lascala A., Martino C., Ragusa S., Nucera S., Walker R., Gratteri S., Mollace V. (2016). Molecular mechanisms of lipid- and glucose-lowering activities of bergamot flavonoids. Pharma Nutr..

[19] Janda E., Salerno R., Martino C., Lascala A., La Russa D., Oliverio M. (2018). Qualitative and quantitative analysis of the proautophagic activity of citrus flavonoids from bergamot polyphenol fraction. Data Brief.

[20] Lascala A., Martino C., Parafati M., Salerno R., Oliverio M., Pellegrino D., Mollace V., Janda E. (2018). Analysis of proautophagic activities of citrus flavonoids in liver cells reveals the superiority of a natural polyphenol mixture over pure flavones. J. Nutr. Biochem..

[21] Lee Y.S., Cha B.Y., Choi S.S., Choi B.K., Yonezawa T., Teruya T., Nagai K., Woo J.T. (2013). Nobiletin improves obesity and insulin resistance in high-fat diet-induced obese mice. J. Nutr. Biochem..

[22] Park H.J., Jung U.J., Cho S.J., Jung H.K., Shim S., Choi M.S. (2013). Citrus unshiu peel extract ameliorates hyperglycemia and hepatic steatosis by altering inflammation and hepatic glucose- and lipid-regulating enzymes in db/db mice. J. Nutr. Biochem..

[23] Gliozzi M., Walker R., Muscoli S., Vitale C., Gratteri S., Carresi C., Musolino V., Russo V., Janda E., Ragusa S (2013). Bergamot polyphenolic fraction enhances rosuvastatin-induced effect on ldl-cholesterol, lox-1 expression and protein kinase b phosphorylation in patients with hyperlipidemia. Int. J. Cardiol..

[24] Mollace V., Sacco I., Janda E., Malara C., Ventrice D., Colica C., Visalli V., Muscoli S., Ragusa S., Muscoli C. (2011). Hypolipemic and hypoglycaemic activity of bergamot polyphenols: From animal models to human studies. Fitoterapia.

[25] Ehrlich J., Gliozzi M., Janda E., Walker R., Romeo F., Mollace V. (2014). Effect of citrus bergamot polyphenol extract on patients with nonalcoholic fatty liver disease. Am. J. Gastroenterol..

[26] Navarra M., Mannucci C., Delbo M., Calapai G. (2015). Citrus bergamia essential oil: From basic research to clinical application. Front. Pharmacol..

[27] Spigoni V., Mena P., Fantuzzi F., Tassotti M., Brighenti F., Bonadonna R.C., Del Rio D., Dei Cas A. (2017). Bioavailability of bergamot (citrus bergamia) flavanones and biological activity of their circulating metabolites in human pro-angiogenic cells. Nutrients.

[28] Salerno R., Casale F., Calandruccio C., Procopio A. (2016). Characterization of flavonoids in citrus bergamia (Bergamot) polyphenolic fraction by liquid chromatography–high resolution mass spectrometry (LC/HRMS). Pharma Nutr..

[29] Leopoldini M., Malaj N., Toscano M., Sindona G., Russo N. (2010). On the inhibitor effects of bergamot juice flavonoids binding to the 3-hydroxy-3-methylglutaryl-coa reductase (hmgr) enzyme. J. Agric. Food Chem..

[30] Walker R., Janda E., Mollace V. (2014). Chapter 84—The use of bergamot-derived polyphenol fraction in cardiometabolic risk prevention and its possible mechanisms of action. Polyphen Hum. Health Dis..

[31] Impellizzeri D., Bruschetta G., Di Paola R., Ahmad A., Campolo M., Cuzzocrea S., Esposito E., Navarra M. (2015). The anti-inflammatory and antioxidant effects of bergamot juice extract (Bje) in an experimental model of inflammatory bowel disease. Clin. Nutr..

[32] Impellizzeri D., Cordaro M., Campolo M., Gugliandolo E., Esposito E., Benedetto F., Cuzzocrea S., Navarra M. (2016). Anti-inflammatory and antioxidant effects of flavonoid-rich fraction of bergamot juice (Bje) in a mouse model of intestinal ischemia/reperfusion injury. Front. Pharmacol..

[33] Promrat K., Kleiner D.E., Niemeier H.M., Jackvony E., Kearns M., Wands J.R., Fava J.L., Wing R.R. (2010). Randomized controlled trial testing the effects of weight loss on nonalcoholic steatohepatitis. Hepatology.

[34] Khan H.A. (2003). Calcdose: A software for drug dosage conversion using metabolically active mass of animals. Drug Chem. Toxicol..

[35] Antunes L.C., Elkfury J.L., Jornada M.N., Foletto K.C., Bertoluci M.C. (2016). Validation of homa-ir in a model of insulin-resistance induced by a high-fat diet in wistar rats. Arch. Endocrinol. Metab..

[36] Daniels S.J., Leeming D.J., Eslam M., Hashem A.M., Nielsen M.J., Krag A., Karsdal M.A., Grove J.I., Guha I.N., Kawaguchi T. (2018). Adapt: An algorithm incorporating PRO-C3 accurately identifies patients with nafld and advanced fibrosis. Hepatology.

[37] Asgharpour A., Cazanave S.C., Pacana T., Seneshaw M., Vincent R., Banini B.A., Kumar D.P., Daita K., Min H.K., Mirshahi F. (2016). A diet-induced animal model of non-alcoholic fatty liver disease and hepatocellular cancer. J. Hepatol..

[38] Peinnequin A., Mouret C., Birot O., Alonso A., Mathieu J., Clarencon D., Agay D., Chancerelle Y., Multon E. (2004). Rat pro-inflammatory cytokine and cytokine related mrna quantification by real-time polymerase chain reaction using sybr green. BMC Immunol..

[39] Lackner C. (2011). Hepatocellular ballooning in nonalcoholic steatohepatitis: The pathologist’s perspective. Expert Rev. Gastroenterol. Hepatol..

[40] Williams K.H., Shackel N.A., Gorrell M.D., McLennan S.V., Twigg S.M. (2013). Diabetes and nonalcoholic fatty liver disease: A pathogenic duo. Endocr. Rev..

[41] Bravo E., Palleschi S., Aspichueta P., Buque X., Rossi B., Cano A., Napolitano M., Ochoa B., Botham K.M. (2011). High fat diet-induced non alcoholic fatty liver disease in rats is associated with hyperhomocysteinemia caused by down regulation of the transsulphuration pathway. Lipids Health Dis..

[42] Roza N.A., Possignolo L.F., Palanch A.C., Gontijo J.A. (2016). Effect of long-term high-fat diet intake on peripheral insulin sensibility, blood pressure, and renal function in female rats. Food Nutr. Res..

[43] Amato A., Caldara G.F., Nuzzo D., Baldassano S., Picone P., Rizzo M., Mule F., Di Carlo M. (2017). Nafld and atherosclerosis are prevented by a natural dietary supplement containing curcumin, silymarin, guggul, chlorogenic acid and inulin in mice fed a high-fat diet. Nutrients.

[44] Belemets N., Kobyliak N., Virchenko O., Falalyeyeva T., Olena T., Bodnar P., Savchuk O., Galenova T., Caprnda M., Rodrigo L. (2017). Effects of polyphenol compounds melanin on nafld/nash prevention. Biomed. Pharmacother..

[45] Kim B., Farruggia C., Ku C.S., Pham T.X., Yang Y., Bae M., Wegner C.J., Farrell N.J., Harness E., Park Y.K. (2017). Astaxanthin inhibits inflammation and fibrosis in the liver and adipose tissue of mouse models of diet-induced obesity and nonalcoholic steatohepatitis. J. Nutr. Biochem..

[46] Morrison M.C., Liang W., Mulder P., Verschuren L., Pieterman E., Toet K., Heeringa P., Wielinga P.Y., Kooistra T., Kleemann R. (2015). Mirtoselect, an anthocyanin-rich bilberry extract, attenuates non-alcoholic steatohepatitis and associated fibrosis in ApoE(*)3Leiden mice. J. Hepatol..

[47] Seo E., Oh Y.S., Jun H.S. (2016). *Psoralea corylifolia* L. Seed extract attenuates nonalcoholic fatty liver disease in high-fat diet-induced obese mice. Nutrients.

[48] Glass L.M., Dickson R.C., Anderson J.C., Suriawinata A.A., Putra J., Berk B.S., Toor A. (2015). Total body weight loss of >/= 10% is associated with improved hepatic fibrosis in patients with nonalcoholic steatohepatitis. Dig. Dis. Sci..

[49] Cheng D.M., Pogrebnyak N., Kuhn P., Poulev A., Waterman C., Rojas-Silva P., Johnson W.D., Raskin I. (2014). Polyphenol-rich rutgers scarlet lettuce improves glucose metabolism and liver lipid accumulation in diet-induced obese c57bl/6 mice. Nutrition.

[50] Chang H.C., Peng C.H., Yeh D.M., Kao E.S., Wang C.J. (2014). Hibiscus sabdariffa extract inhibits obesity and fat accumulation, and improves liver steatosis in humans. Food Funct..

[51] Chang W.C., Jia H., Aw W., Saito K., Hasegawa S., Kato H. (2014). Beneficial effects of soluble dietary jerusalem artichoke (Helianthus tuberosus) in the prevention of the onset of type 2 diabetes and non-alcoholic fatty liver disease in high-fructose diet-fed rats. Br. J. Nutr..

[52] Ham J.R., Lee H.I., Choi R.Y., Sim M.O., Seo K.I., Lee M.K. (2016). Anti-steatotic and anti-inflammatory roles of syringic acid in high-fat diet-induced obese mice. Food Funct..

[53] Barr V.A., Malide D., Zarnowski M.J., Taylor S.I., Cushman S.W. (1997). Insulin stimulates both leptin secretion and production by rat white adipose tissue. Endocrinology.

[54] Lee M.J., Fried S.K. (2006). Multilevel regulation of leptin storage, turnover, and secretion by feeding and insulin in rat adipose tissue. J. Lipid Res..

[55] Cohen B., Novick D., Rubinstein M. (1996). Modulation of insulin activities by leptin. Science.

[56] Perez-Cano F.J., Castell M. (2016). Flavonoids, inflammation and immune system. Nutrients.

[57] Ferlazzo N., Cirmi S., Calapai G., Ventura-Spagnolo E., Gangemi S., Navarra M. (2016). Anti-inflammatory activity of citrus bergamia derivatives: Where do we stand?. Molecules.

[58] Mulvihill E.E., Burke A.C., Huff M.W. (2016). Citrus flavonoids as regulators of lipoprotein metabolism and atherosclerosis. Annu. Rev. Nutr..

[59] Ferreira P.S., Spolidorio L.C., Manthey J.A., Cesar T.B. (2016). Citrus flavanones prevent systemic inflammation and ameliorate oxidative stress in C57BL/6J mice fed high-fat diet. Food Funct..

[60] Felgines C., Texier O., Morand C., Manach C., Scalbert A., Regerat F., Remesy C. (2000). Bioavailability of the flavanone naringenin and its glycosides in rats. Am. J. Physiol. Gastrointest. Liver Physiol..

[61] Kay C.D., Pereira-Caro G., Ludwig I.A., Clifford M.N., Crozier A. (2017). Anthocyanins and flavanones are more bioavailable than previously perceived: A review of recent evidence. Annu. Rev. Food Sci. Technol..

[62] Akiyoshi H., Terada T. (1999). Centrilobular and perisinusoidal fibrosis in experimental congestive liver in the rat. J. Hepatol..

[63] Tokunaga Y., Osawa Y., Ohtsuki T., Hayashi Y., Yamaji K., Yamane D., Hara M., Munekata K., Tsukiyama-Kohara K., Hishima T. (2017). Selective inhibitor of Wnt/beta-catenin/CBP signaling ameliorates hepatitis C virus-induced liver fibrosis in mouse model. Sci. Rep..

[64] Lozano I., Van der Werf R., Bietiger W., Seyfritz E., Peronet C., Pinget M., Jeandidier N., Maillard E., Marchioni E., Sigrist S. (2016). High-fructose and high-fat diet-induced disorders in rats: Impact on diabetes risk, hepatic and vascular complications. Nutr. Metab..

[65] Zeeni N., Dagher-Hamalian C., Dimassi H., Faour W.H. (2015). Cafeteria diet-fed mice is a pertinent model of obesity-induced organ damage: A potential role of inflammation. Inflamm. Res..

