# Supplementary material for: Bergamot Polyphenols Boost Therapeutic Effects of the Diet on Non-Alcoholic Steatohepatitis (NASH) Induced by “Junk Food”: Evidence for Anti-Inflammatory Activity
Source: Nutrients. 2018 Nov 1;10(11):1604. doi: 10.3390/nu10111604 (PMC6267059; doi:10.3390/nu10111604)
Supplement: Supplementary file 1 [file nutrients-10-01604-s001.pdf]

nutrients

Supplementary material

Bergamot Polyphenols Boost Therapeutic Effects of the Diet on Non-Alcoholic Steatohepatitis (NASH) Induced by "Junk Food": Evidence for Anti-Inflammatory Activity

Maddalena Parafati [1,2,†], Antonella Lascala [1,2,†], Daniele La Russa [1,3], Chiara Mignogna [4], Francesca Trimboli [1], Valeria Maria Morittu [1], Concetta Riillo [1], Rachele Macirella [3], Vincenzo Mollace [1,2], Elvira Brunelli [3] and Elzbieta Janda [1,2,*]

[1] Department of Health Sciences, Magna Graecia University, Campus Germaneto, 88100 Catanzaro, Italy; mparafati@unicz.it (M.P.); anto.lascala@gmail.com (A.L.); dlarussa@hotmail.it (D.L.R.); trimboli@unicz.it (F.T.); morittu@unicz.it (V.M.M.); criillo@unicz.it (C.R.); mollace@unicz.it (V.M.)
[2] Interregional Research Center for Food Safety and Health, 88100 Catanzaro, Italy
[3] Department of Biology, Ecology and Earth Sciences, University of Calabria, 87036 Rende (CS), Italy; rachele.macirella@unical.it (R.M.); elvira.brunelli@unical.it (E.B.)
[4] Department of Experimental and Clinical Medicine, Magna Graecia University, Campus Germaneto, 88100 Catanzaro, Italy; mignogna@unicz.it
* Correspondence: janda@unicz.it
† Both authors contributed equally to this work.

Table of contents

Supplementary Table 1 ……………………………………………………..........................2

Supplementary Fig. 1………………………………………………….............................3

Supplementary Fig. 2 ………………………………………………….............................4

Supplementary Fig. 3………………………………………………….............................5

Supplementary Table S1.

List of the food items used to assemble *Cafeteria* (CAF) diet and related nutritional values. One sweet and one salty snack was provided at libitum and supplemented every 4-5 days. Harlan 2016 diet was also provided at libitum to all experimental groups.

Food item/Nutritional content (in 1 g)	Kcal	Fats*	Saturated fats*	Proteins*	Total carbo-hydrates*	Sugar*	Fiber*
Harlan 2016	3.00	0.04	0.01	0.16	0.64	0.05	0.04
Chocolate snack 1	5.86	0.39	0.16	0.08	0.49	0.42	0.03
Breakfast cereals with chocolate A	3.86	0.03	0.02	0.06	0.82	0.29	n.d.
Potato Chips 1	5.22	0.34	0.05	0.04	0.51	0.01	0.03
Cracker with cheese cream flavored filling	5.17	0.28	0.10	0.10	0.54	0.05	0.03
Snickers (Chocolate snack 2)	4.84	0.23	0.08	0.09	0.60	0.52	0.00
Tortilla chips	5.00	0.23	n.d.	0.07	0.65	n.d.	n.d.
Provola (Italian cheese)	2.92	0.21	0.14	0.26	0.01	0.01	0.00
TUC bacon	4.90	0.23	0.17	0.08	0.62	0.07	0.03
Smoked chopped bacon 1	3.13	0.25	0.07	0.22	0.00	0.00	0.00
Potato Chips-2	5.67	0.35	n.d.	0.06	0.57	n.d.	n.d.
Chocolate snack whith milk cream filling	5.52	0.34	0.20	0.08	0.53	0.53	0.01
Mortadella (Italian cured sausage)	3.08	0.28	n.d.	0.13	0.01	0.00	0.00
Provolone dolce (Italian cheese)	3.40	0.26	0.18	0.26	0.01	0.00	0.00
Grana Padano (Italian cheese)	3.84	0.28	n.d	0.33	n.d	n.d.	n.d.
Canestrelli (butter cookies)	5.28	0.27	n.d.	0.07	0.64	n.d.	n.d.
Croccantelle (crackers) Bacon flavoured	1.54	0.06	0.01	0.06	0.03	0.00	0.00
Breakfast cereals with chocolate B	3.76	0.03	0.01	0.08	0.78	0.25	0.05
Danish Butter & Chocolate Chip Cookies	5.00	0.24	n.d.	0.06	0.65	n.d.	0.02
Potato Chips 3	5.43	0.33	0.04	0.05	0.55	0.01	0.03
Snack with potatoes and cheese	5.16	0.26	0.13	0.07	0.63	0.04	0.02
Würstel 1	2.36	0.18	0.05	0.11	0.05	0.01	0.00
Provolone piccante (Italian cheese)	4.04	0.32	0.22	0.29	0.00	0.00	0.00
Concentrate milk with added sugar	3.22	0.08	0.05	0.07	0.55	0.55	0.00
Peanuts, toasted and salted	6.02	0.53	0.08	0.25	0.07	0.04	0.07
Milk cream, UHT treated	2.11	0.21	0.14	0.03	0.04	0.04	0.00
Breakfast cereals with chocolate C	3.80	0.03	0.02	0.11	0.75	0.28	0.07
Potato Chips 4	5.46	0.33	0.04	0.07	0.54	0.00	0.04
Muffin with hazelnut cream	4.29	0.22	n.d.	0.06	0.52	n.d.	n.d.
Muffin with chocolate cream 1	4.45	0.23	n.d.	0.06	0.53	n.d.	n.d.
Würstel 2	3.48	0.32	0.13	0.14	0.01	0.01	0.00
Cracker Krit	5.04	0.26	n.d.	0.06	0.61	n.d.	n.d.
Chopped bacon	3.51	0.31	n.d.	0.18	0.01	n.d.	n.d.
Muffin with chocolate cream 2	3.93	0.18	n.d.	0.05	0.53	n.d.	n.d.

* The contents are expressed in g per 1 g of food item

Figure S1

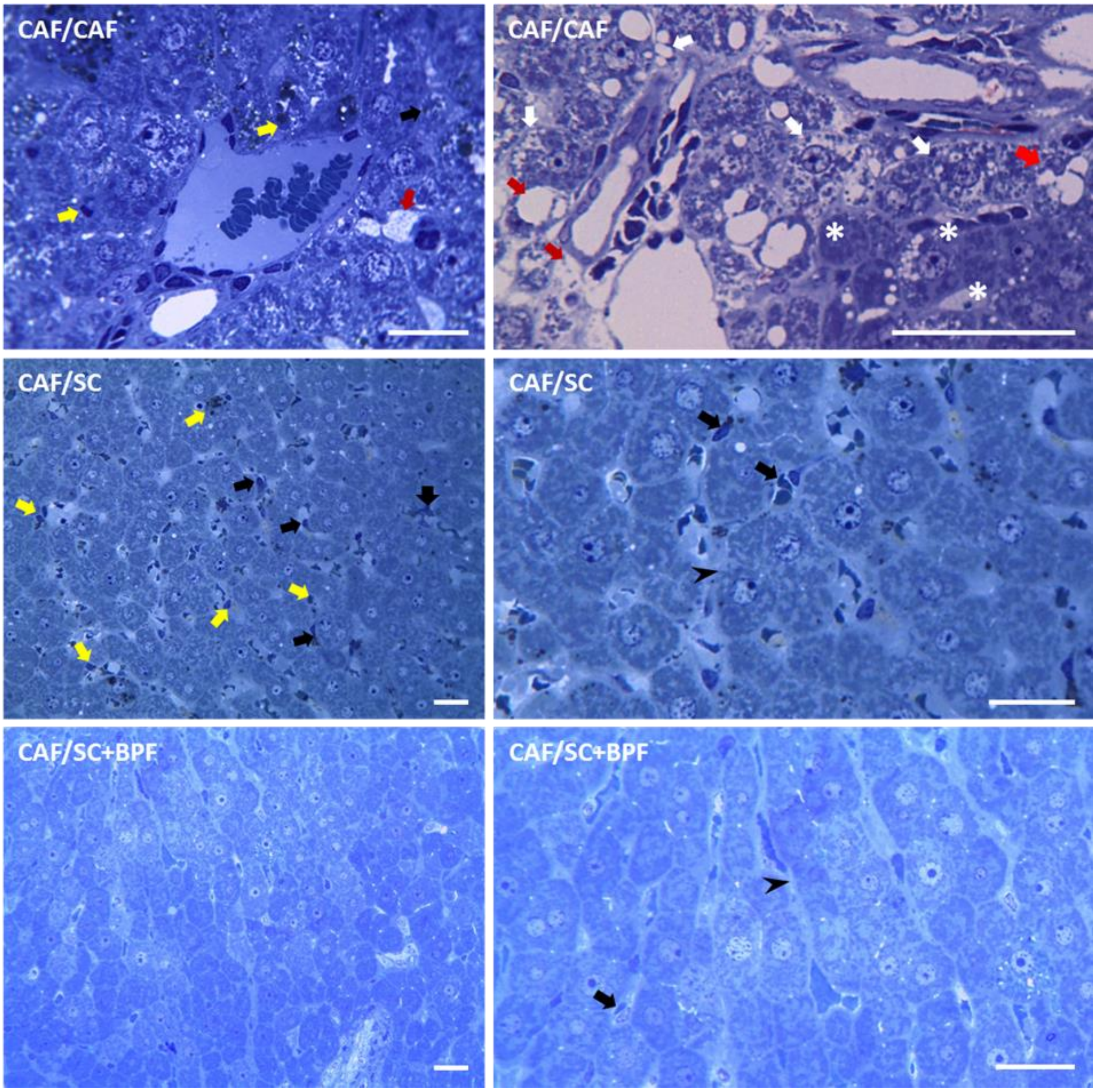

Figure S1. **Toluidine-blue staining of representative liver sections from 3 different treatment groups: CAF/CAF (16 + 10 weeks), CAF/SC, and CAF/SC+BPF**. Arrowhead= sinusoids; black arrow= Kupffer cells; yellow arrow= lymphocytes; white arrow= vesicular degeneration; red arrow= hepatocyte ballooning; asterisk= glycogen granules. All scale bars= 25 μm.

Figure S2

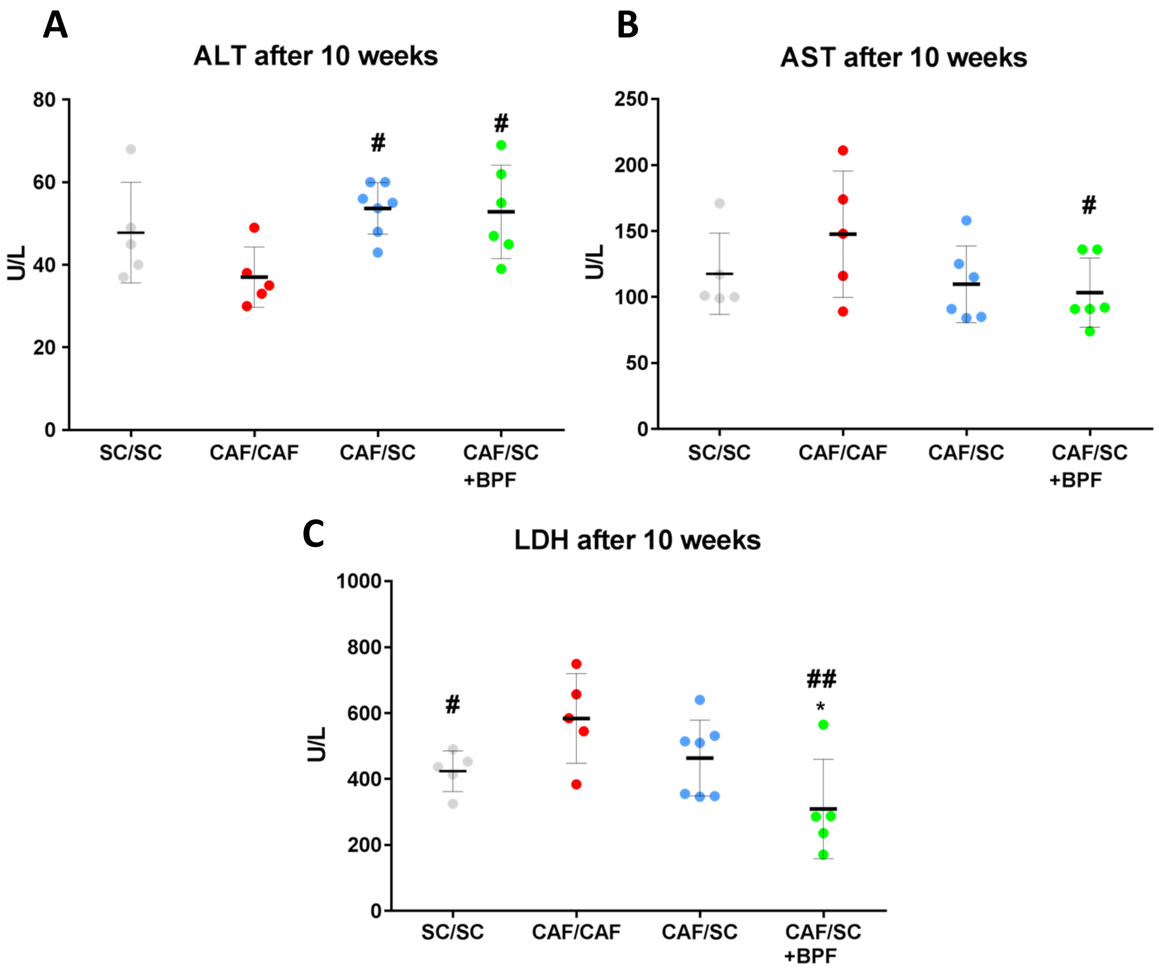

Figure S2. Evaluation of serum biochemical parameters of liver injury. A) Alanine aminotransferase (ALT), B) Aspartate transaminase (AST), and C) Lactate dehydrogenase (LDH) levels from 3 different treatment groups. Data are represented as means ± SD (5 is the minimum number of animals assigned to different experimental groups). For statistical analysis t-test was performed. ALT, # p≤0.01 denotes differences statistically significant CAF/CAF vs CAF/SC and CAF/SC+BPF; AST, # p≤,0.04 CAF/CAF vs CAF/S+BPFC; and LDH, # p≤,0.02 CAF/CAF vs SC/SC; ## p≤,0.008 CAF/CAF vs CAF/SC+BPF; *p≤,0.03 SC/SC vs CAF/SC+BPF.

Figure S3

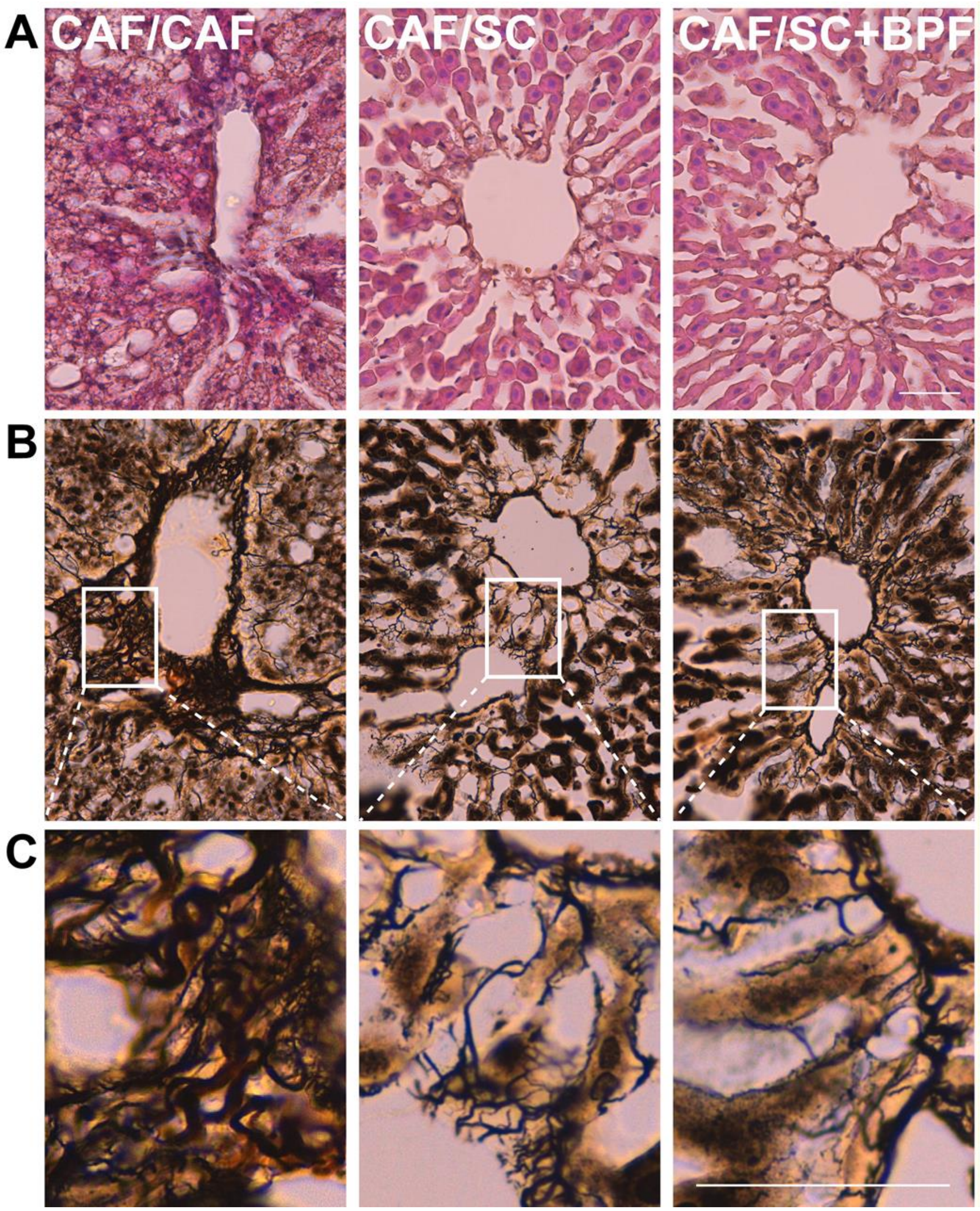

Figure S3. Histological analysis of liver fibrosis in Wistar rats exposed to CAF diet for 26 weeks (CAF/CAF) or exposed to CAF diet for 16 weeks and then to SC or SC+BPF diet for 10 weeks. A) HE stained sections of the portal region of paraffin-embedded liver samples. B) Silver impregnation (SI) staining revealing reticular fibers. C) A magnified region of the section in B. All scale bars 50 µm. Note extensive areas of thick fibers around the blood vessels of the portal region in CAF/CAF liver sections and only rare and thin fibers in the hepatic tissue of the CAF/SC and CAF/SC+BPF sections. These sections show only few thick fibers directly adjacent to the lumen of the blood vessels corresponding to the endothelial layer. Braun diffuse areas inside hepatocytes: unspecific SI staining.
